# Restrictive parenteral fluid therapy in infants and children presenting with acute severe viral pneumonia in the PICU: a single-center experience

**DOI:** 10.1186/cc14047

**Published:** 2014-12-03

**Authors:** S Kandasamy, N Vijayakumar, T Sankaralingam, V Varadarajan, N Krishnamoorthy

**Affiliations:** 1 Division of Pediatric Intensive Care, Department of Pediatrics, Mehta Children's Hospital Pvt Ltd, Chennai, India

## Introduction

Acute viral (RSV and non-RSV) pneumonia is one of the most common causes of lower respiratory tract infection in infants and children. Although only 2 to 3% of infants with bronchiolitis require hospitalized management based on published literature, in recent years we see more children requiring hospitalization in high-dependency units. Most of these children require intravenous fluid therapy and some form of oxygen supplementation. As there is no standardized evidence-based parenteral fluid therapy protocol specific for acute severe viral pneumonia available, we decided to retrospectively analyze the result of our restrictive fluid therapy protocol.

## Methods

All children less than 5 years age admitted to our PICU from June 2013 to December 2013 with etiological evaluation confirmed acute severe viral (RSV and non-RSV) pneumonia who received a minimum 48 hours of parenteral fluid therapy were enrolled and the data retrieved from the case sheet. The data analyzed were duration of PICU stay, hospital stay, oxygen therapy requirement, duration of oxygen therapy and their relationship to fluid balance on restrictive fluid regimen.

## Results

A total of 32 children met the criteria for inclusion in the study period, 23 boys (71.9%) and nine girls (28.1%). The median age of children was 9.5 months (IQR 3.3 to 27.3). Of 32 children, 4/32 (12.5%) required invasive mechanical ventilation; 5/32 (15.6%) required NP-CPAP; 12/32 (37.5%) were on HHFNC; and nasal prong oxygen supplementation in 26 (81.3%) children. The median duration of ICU stay was 68.9 hours (IQR 39.2 to 80.2), that of hospital stay was 116.5 (IQR 92 to 179.2). Fluid balance at the end of 72 hours of ICU stay did not significantly differ the need for ventilation (*P *= 0.45) or the duration of ventilation (*P *= 0.60). Fluid balance in the first 72 hours correlated positively with change in serum sodium levels indicating a fall in sodium levels with more positive fluid balance (Spearman coefficient of 0.452 (*P *= 0.16)). There was no significant correlation between fluid balance and duration of PICU/hospital stay (*P *= 0.58/0.75). eGFR calculated using modified Schwartz formula did not correlate with treatment parameters like duration of ICU/hospital stay, mechanical ventilation, and CPAP.

## Conclusion

Positive fluid balance may not influence the duration of PICU stay, hospital stay, or oxygen therapy in children with severe viral pneumonia when initiated on restrictive (70% of Holliday Segar calculation) parenteral fluid therapy. eGFR at admission did not influence the fluid balance when they were uniformly initiated on restrictive parenteral fluids. Further studies with calculated sample size may help to confirm the observations.

